# Genome editing-mediated knock-in of therapeutic genes ameliorates the disease phenotype in a model of hemophilia

**DOI:** 10.1016/j.omtn.2022.08.002

**Published:** 2022-08-04

**Authors:** Jeong Hyeon Lee, Hye-Kyung Oh, Beom Seok Choi, Ho Hyeon Lee, Kyu Jun Lee, Un Gi Kim, Jina Lee, Hyerim Lee, Geon Seong Lee, Se Jun Ahn, Jeong Pil Han, Seokjoong Kim, Su Cheong Yeom, Dong Woo Song

**Affiliations:** 1 Graduate School of International Agricultural Technology and Institute of Green-Bio Science and Technology, Seoul National University, 1447 Pyeongchang-Ro, Daewha, Pyeongchang, Gangwon 25354, Korea; 2 Research and Development Center, Toolgen Incorporated, Geumcheon-gu, Seoul 08501, Korea; 3 WCU Biomodulation Major, Department of Agricultural Biotechnology, Seoul National University, Gwanank-gu, Seoul 08826, Korea

**Keywords:** MT: RNA/DNA editing, adeno-associated virus, apolipoprotein C3, hemophilia, genome editing, knock-in, Campylobacter jejuni Cas9

## Abstract

Recently, clinical trials of adeno-associated virus-mediated replacement therapy have suggested long-term therapeutic effects for several genetic diseases of the liver, including hemophilia. However, there remain concerns regarding decreased therapeutic effects when the liver is regenerated or when physiological proliferation occurs. Although genome editing using the clustered regularly interspaced short palindromic repeats/Cas9 system provides an opportunity to solve this problem, low knock-in efficiency may limit its application for therapeutically relevant expression. Here, we identified a novel gene, *APOC3*, in which a strong promoter facilitated the expression of knocked-in genes in hepatocytes. We also investigated the effects of *APOC3* editing using a small Cas9 protein derived from *Campylobacter jejuni* (CjCas9) in a hemophilic model. We demonstrated that adeno-associated virus-mediated delivery of CjCas9 and donor led to moderate levels of human factor 9 expression in APOC3-humanized mice. Moreover, knock-in-driven expression induced substantial recovery of clotting function in mice with hemophilia B. There was no evidence of off-target editing *in vitro* or *in vivo*. Collectively, our findings demonstrated therapeutically relevant expression using a precise and efficient *APOC3*-editing platform, providing insights into the development of further long-term therapeutics for diverse monogenic liver diseases.

## Graphical abstract

**Figure:**
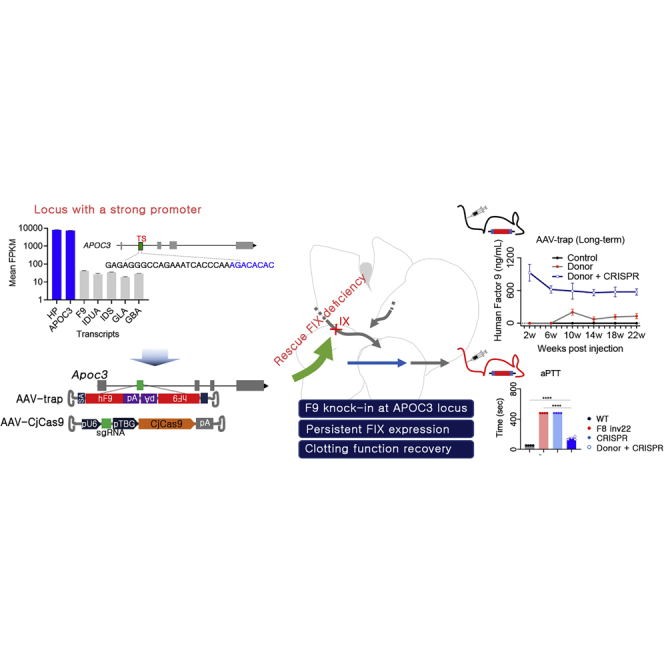

## Teaser

Lee JH et al. established an *in vivo* therapeutic platform for liver diseases. The platform is based on knock-in of a transgene into the specific genomic loci with a strong promoter. The platform produced therapeutically relevant levels of human FIX and promoted functional recovery in the hemophilic model.

## Introduction

Hemophilia B is a monogenic bleeding disorder that has been an interesting target for gene therapy because simple augmentation of the causative gene *F9* using adeno-associated virus (AAV) may be suitable.^1^ Several clinical studies using AAV have demonstrated long-term therapeutic effects. Because this effect may involve human factor IX (hFIX) expression from an episomally stable viral genome, the non-integrated viral genome can be diluted, during which the normal hepatocyte turnover rate was approximately 200 days in a mouse adult liver.^2^ Importantly, such a decrease in therapeutic outcomes can be vulnerable when the adult liver is damaged and regenerating or during physiological proliferation of the liver after birth through adulthood. Therefore, AAV therapy should consider the quality of patient life after treatment and is not effective for neonatal or pediatric patients with liver diseases.^3^ Although lentiviral therapy may overcome such limitations, this approach has still not been validated for safety with regard to random integration or inflammation *in vivo*; thus, almost all clinical trials using lentiviruses have focused on *ex vivo* therapies combined with hematopoietic stem cells (HSCs) or T cells.^4^ Genome editing using programmable nucleases is a powerful tool for specific editing of the target DNA, and the simplicity and efficiency of editing using the recently developed CRISPR/Cas9 system may provide opportunities for long-term therapeutics.^5^ Importantly, persistence of CRISPR/Cas9-induced effects have been extensively studied in liver of preclinical models,^6^^,^^7^ or even in highly proliferating and clinically applied cells, including HSCs or T lymphocytes.^8^^,^^9^ Accordingly, it could be possible that the newly edited genome is be maintained during proliferation by replication of the edited sequence.

CRISPR/Cas9-mediated therapeutic approaches have diverse effects, including silencing of disease-causing proteins, correction of pathogenic mutations, and expression of therapeutic proteins.^5^ Homologous recombination (HR)-mediated knock-in is necessary for the correction of mutations or the expression of therapeutic proteins; however, knock-in approaches are usually less efficient than knock-out approaches.^5^^,^^10^^,^^11^ Although recently developed base editing^12^ or prime editing^13^ may be effective alternatives to HR, these approaches should be applied for editing only one or a few base sequences. Thus, these approaches are not suitable for the insertion of an entire cDNA sequence, which is required in most gene-deficient patients, regardless of the mutation type or location. One possible idea is to generate transgene knock-in at safe harbor loci such as ROSA26 or *AAVS1*, but the approach needs additional exogenous promoter sequences such as CBA or cytomegalovirus (CMV); moreover, the expression from the *AAVS1* was reported to be silenced owing to DNA methylation and other unknown factors.^14^^,^^15^ Alternatively, a specialized genomic loci, whose transcriptional activity is markedly high in specific cells of interest, can be used to overcome the lack of knock-in efficiency and long-term effect.^16^ By using endogenous strong promoter activity, knock-in of transgenes may be used to successfully express the encoded proteins to therapeutically relevant levels.^17^^,^^18^ Importantly, this system could be a versatile platform for applications in various diseases by simply changing the target genes in the donor template. In the liver in particular, hundreds of monogenic diseases have been reported, most of which are still not treatable.^19^ Therefore, there is an urgent need to develop diverse liver-specific genome editing platforms that can assist in treating liver-related diseases, but such advanced approaches have not yet been well established.

In the current study, we developed a novel genome editing therapeutic platform that included two major interesting features. First, the apolipoprotein C3 (*APOC3*) gene was selected as the genomic target site. Since APOC3 possesses a strong transcriptional activity in liver compared with other genetic disease-related genes, knocked-in hepatocytes could express sufficient levels of therapeutic proteins by using relatively strong endogenous expression system. In addition, human genetic studies have suggested that functional loss of APOC3 is not associated with any medical risk and can even be beneficial in modulating cholesterol re-cycling.^20^, ^21^, ^22^ Thus, APOC3 could be an adequate genomic locus for liver-targeting therapeutics in the context of efficiency and safety. Second, small Cas9 derived from *Campylobacter jejuni* (CjCas9), was used, which has entirely different protospacer-adjacent motif (PAM) and sgRNA scaffold sequences from those of the widely used *Streptococcus pyogenes* Cas9 (SpCas9).^23^ The specificity of CjCas9 has been reported to be greater than SpCas9 in previous unbiased off-target studies.^23^^,^^24^ The greater specificity could be also explained by its longer NNNNRYAC PAM than NGG for SpCas9. Such relatively longer and restricted PAM result in more specific genome editing by limiting the number of off-targets by any given guide RNA, similar to that suggested for StCas9 that consist of NNAGAAW or NGGNG PAM.^25^ Additionally, the smaller size of CjCas9 (2.95 kb; 984 amino acids) relative to that of SpCas9 (4.1 kb; 1,368 amino acids), is adaptable for application of diverse optimization elements including tissue specific promoters, enhancers and untranslated regions, and can be effectively packaged with the AAV elements wherein the packaging capacity is limited to 4.7 kb.^23^^,^^26^ For the reasons mentioned above, we selected CjCas9 owing to its relatively high compatibility with the AAV system, which has been safely and effectively used in a number of gene therapy clinical trials. We demonstrated the feasibility of our genome editing strategy for therapeutically relevant expression of hFIX in genetic mouse model for hemophilia B. Therefore, this study established knock-in-mediated therapeutic platforms with potential extension to other liver genetic diseases.

## Results

### Genome editing with strong promoters

**Figure 1:**
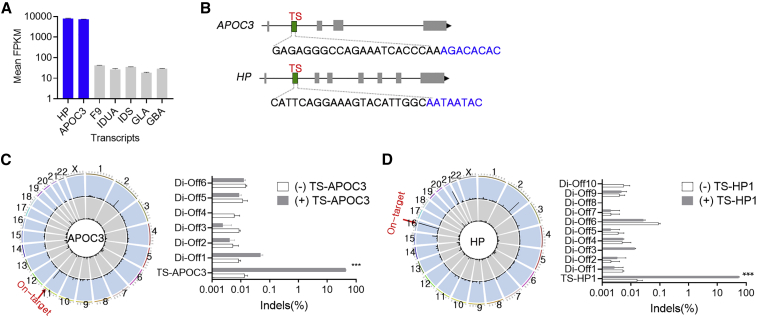
Genome editing of genes with strong promoters (A) RNA sequencing results from human primary hepatocytes (n = 3). (B) A schematic drawing of *APOC3* and *HP* genes and the lead sgRNAs. Green regions in intron 1 of *APOC3* and *HP* genes indicate target sites for the corresponding lead sgRNA, target site (TS)-APOC3 and TS-HP. TS-APOC3 and TS-HP are the same as TS7 and TS10, respectively in Figure S1. Blue text indicates the PAM. (C and D) Left, Genome-wide Circos plots showing *in vitro* cleavage sites in the human genome in the absence (gray) or presence (blue) of TS-APOC3 (C) or TS-HP sgRNA (D). On-target cleavage is indicated by the red arrow. Right, Targeted deep sequencing results for the off-target candidates obtained by Digenome-seq in TS-APOC3- (C) or TS-HP-treated HEK293 cells (D) (n = 3). Data are presented as means ± SEM, and statistical analysis was performed using the Student t test (A), (C), and (D). ∗∗∗p < 0.001.

To use endogenous loci to express transgenes in human hepatocytes, we attempted to uncover novel genes with strong promoter by RNA sequencing from human primary hepatocytes. The results demonstrated that the reads per kilobase of transcript, per million mapped reads values of two genes encoding secreted proteins, i.e., *APOC3* and haptoglobin (*HP*), were approximately 200 times higher than those of various genes associated with monogenic diseases, including hemophilia and lysosomal storage diseases (Figure 1A). Using an *in silico* tool, Cas9 Designer (www.rgenome.net), single guide RNA (sgRNA) candidates were identified as the sequences having 22 nucleotides adjacent to the PAM NNNNRYAC in the first intron of human HP or APOC3 gene. Subsequently, we tested the sgRNA activity using transfected plasmids expressing the sgRNAs and CjCas9 in HEK293 cells (Figure 1B), and we found that the sgRNAs TS-APOC3 and TS-HP induced the most efficient insertions and deletions (indels; Figures S1A and S1B). Next, off-target candidates were obtained by a homology-based *in silico* method combined with digenome-seq, which is an unbiased method for detecting off-target sites when the whole genome is incubated with Cas9 and sgRNA *in vitro* (Table S1). In total, eight homologous sites with up to four mismatched nucleotides and six sites found in digenome-seq were subsequently validated for TS-APOC3. Targeted deep sequencing demonstrated no active off-targets when the APOC3 on-target indel rates were 43.6% or 76.6% (Figures 1C and S2). Similarly, we observed that all 10 off-target loci found in digenome-seq were not actively edited by TS-HP1, which had an on-target indel rate of 55.1% (Figure 1D). Although we used HEK293, owing to their high transfection efficiency, the sgRNAs for target cell type such as hepatocyte-like cells, needs to be determined. Thus, we performed similar specificity test in Hep G2 cells using digenome-seq for detecting off-target sites associated for TS-APOC3, six off-target candidates and no active off-target sites were identified, further validating the specificity of TS-APOC3 that can also be demonstrated consistently in the hepatocyte like cell types (Figure S3). Taken together, the results suggested that our lead sgRNAs enabled efficient and precise editing of the genes with a strong promoter.

### Knock-in-driven expression of therapeutic proteins in APOC3-expressing cells

CRISPR-Cas9-induced large deletions have been reported in the context of safety risk,^27^ and unexpected editing can lead to a decrease in protein expression via splicing abnormalities or frameshift mutations. Interestingly, homozygous or heterozygous APOC3 loss-of-function mutations have been reported to have cardioprotective benefits in genome-wide association studies of humans.^20^, ^21^, ^22^ This suggests that genome editing of the APOC3 intron could be a safer approach, even if an unexpected large deletion arises at the on-target site. Thus, we selected APOC3 for further study to validate knock-in-driven expression in cells and mouse livers.

**Figure 2:**
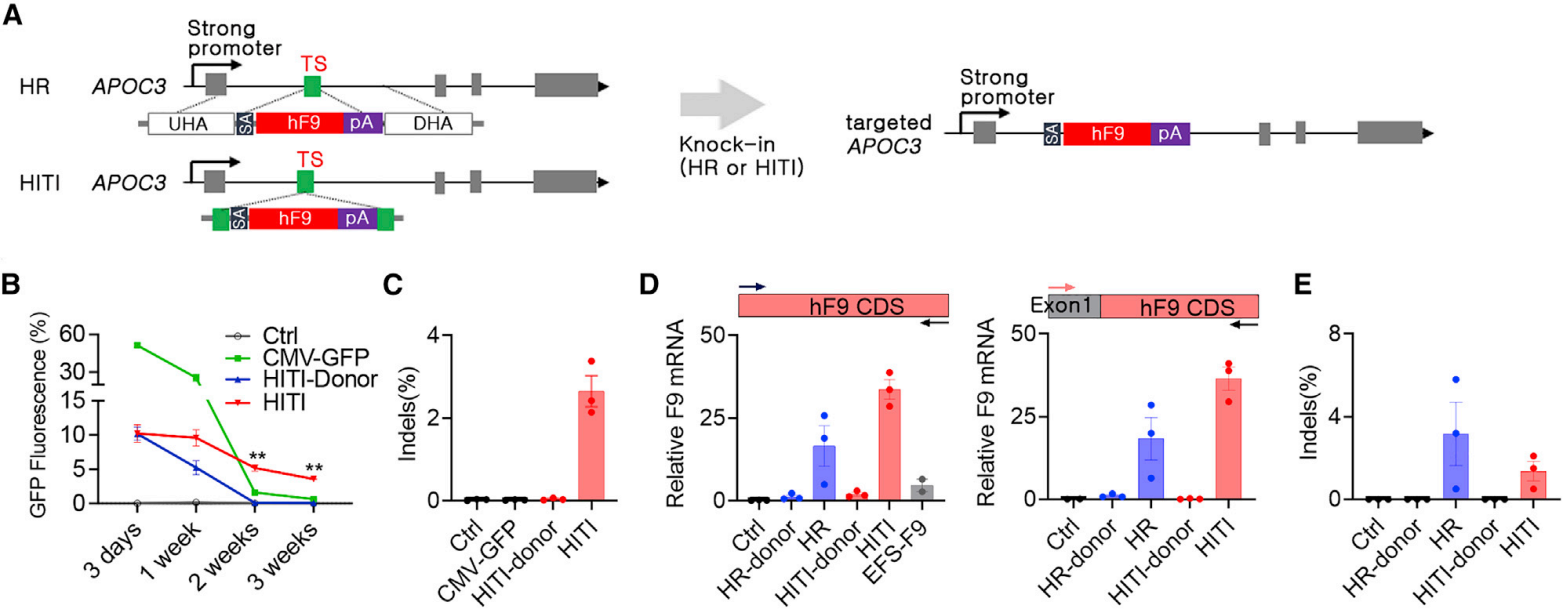
Knock-in-driven expression of therapeutic proteins in APOC3-expressing cells (A) Strategy for genome editing-mediated knock-in of human *F9* in the TS-APOC3 site. Combination of CjCas9 and TS-APOC3 with the HR donor or HITI donor results in the expression of hF9 by the targeted *APOC3* genomic structure after splicing of the knocked-in sequence containing the splicing acceptor (SA) and *F9* and the bovine growth hormone polyadenylation signal (pA). UHR, upstream homology arm; DHR, downstream homology arm. (B) Relative percentages of GFP-positive Hep G2 cells (%) after transfection with GFP-CMV, HITI-donor-only, and HITI (CjCas9 and donor) (n = 4–5). The HITI donor structure is same as in the strategy (A), except that *GFP* cDNA was used instead of *F9* cDNA. Note that only HITI-treated cells showed sustained GFP expression over time. (C) Indel frequencies of cells after FACS analysis at 3 weeks after transfection (n = 4–5). (D and E) *F9* mRNA expression (D) and indel frequency (E) in Hep G2 cells transfected with HR or HITI donor in the absence or presence of the plasmid-expressing CjCas9 and TS-APOC3 (n = 4). Ctrl: untransfected cells. Primer locations for quantitative RT-PCR designed within the F9 CDS (left) or at a junction between *APOC3* exon 1 and the F9 CDS (right) are indicated by arrows (D). Data are presented as means ± SEM, and statistical analysis was performed using the Student t test. ∗∗p < 0.01.

After APOC3 expression was validated in Hep G2 cells, we initially tested transgene expression by transfection of a green fluorescent protein (GFP) donor plasmid in the absence or presence of the plasmid delivering CjCas9 and TS-APOC3. Homology-independent targeted integration (HITI) was initially selected because it has been shown to be an efficient knock-in strategy for dividing and nondividing cells^28^ (Figure 2A). At 3 days after transfection, GFP-positive cells were comparable between donor-only and HITI (CjCas9 and donor). However, at 2–3 weeks after transfection, HITI-transfected cells showed clearly sustained GFP expression, whereas the GFP signal was almost absent in donor only- or CMV-GFP-transfected cells (Figures 2B and 2C). These findings indicate that APOC3 editing-mediated knock-in could induce persistent expression, even in dividing cells in which plasmid-driven expression should be diluted, as observed in the CMV-GFP group. Next, we tested the expression of hFIX using two different knock-in approaches, HITI and HR. The results showed that both HITI and HR led to substantial expression at 2 weeks after transfection (Figure 2D, left). In addition, a similar result was observed with primers targeting endogenous *APOC3* exon 1 and exogenous hFIX (hF9) coding sequence (CDS) (Figure 2D, right), even when the indel frequency was not high (Figure 2E), further indicating that expression was driven by knock-in at the *APOC3* target site. Subsequently, the expression of other transgenes, including *F8* and lysosomal acid glucosylceramidase precursor (*GBA*), was also verified when the same editing platform with the corresponding donor was applied (Figures S4B and S4C). Taken together, these results suggested that genome editing of *APOC3* resulted in efficient expression of diverse transgenes through knock-in of donor DNAs in hepatocyte-like cells.

### Generation of a humanized disease model by embryonic editing

**Figure 3:**
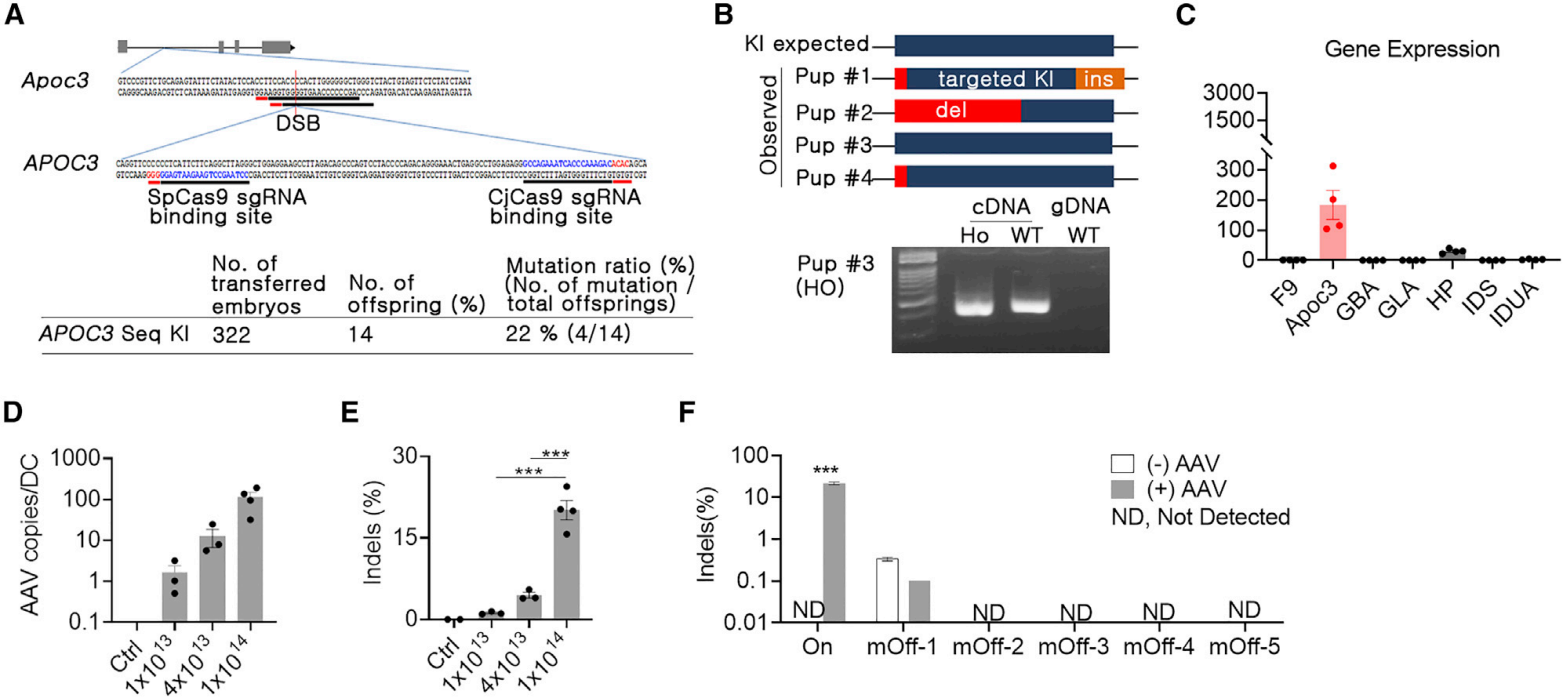
Humanized mouse model and *in vivo* genome editing by AAV delivery (A) Summary of human *APOC3* sequence knock-in mouse generation using SpCas9, two overlapping sgRNAs, and single-stranded oligonucleotides. *Apoc3*, mouse *Apoc3* gene; *APOC3*, human *APOC3* sequence. (B) Sanger sequencing, RT-PCR, and PCR were conducted to confirm KI and alternative splicing. cDNA, cDNA; gDNA, genomic DNA; Ho, homozygote; WT, wild-type. (C) Gene expression comparisons between *Apoc3* and disease-causing target genes using mouse liver tissue by quantitative PCR. (D and E) Quantification of AAV copies per diploid cell (D) and indel frequencies (E) in liver tissues at 6 weeks after infection with AAV8-CjCas9-TS-APOC3 (n = 3–4). Ctrl: AAV-untreated livers were used as control. Total AAV doses (vg/kg) for three treated group are indicated. (F) Off-target results for the top five homologous candidates in the mouse genome. Three livers with higher on-target editing in the high-dose AAV group (+AAV) and were selected and subjected to targeted deep sequencing with three Ctrl livers (–AAV). Note that no active off-target loci were observed. Data are presented as means ± SEM, and statistical analysis was performed using the Student t test. ∗∗∗p < 0.001.

Human-specific TS-APOC3 cannot be used for the mouse genome; therefore, we generated a humanized mouse model in which the human sequence for TS-APOC3 was introduced into the first intron of mouse *Apoc3* by embryonic editing (Figure 3A). Single-stranded oligodeoxynucleotides (ssODNs) were designed with 40-nt homologous arms on both sides and a 120-nt human sequence containing the TS-APOC3 site (Table S2). After electroporation of SpCas9/sgRNA ribonucleoprotein and ssODN into embryos, four of the offspring mice were shown to have the human sequence (Figures 3A and 3B). One mouse contained an intact binding site for TS-APOC3 (Figure 3B, top). There were no abnormalities in reproduction or growth, and there was no evidence of alternative splicing (Figure 3B, bottom). Next, relative *Apoc3* gene expression was analyzed using cDNA from the liver tissue of homozygote APOC3 knock-in mice, and *Apoc3* showed approximately 40- to 300-fold higher expression than other disease-causing genes (Figure 3C). This indicated that the generated humanized mice (B6.Apoc3^APOC3^) could be used to investigate the effects of the APOC3-editing platform in an *in vivo* context.

### AAV-mediated delivery and *in vivo* on-target/off-target editing

To apply our genome editing approach *in vivo*, we examined the optimal AAV delivery conditions in the mouse liver. Among several AAV serotypes with liver tropism, AAV8 infected the mouse liver more efficiently than AAV9, as measured by viral genome copy analysis and GFP immunofluorescence when injected intravenously (Figure S5). Next, we packaged DNA encoding CjCas9 and TS-APOC3 in AAV8, and APOC3-humanized mice were injected with the AAV to observe editing of the human *APOC3* target site. *APOC3* editing was found to be dose dependent, and the dose dependency was consistent with quantification data for the infected viral copy numbers. In high-dose AAV-injected livers (1 × 10^14^ vg/kg), on-target indels were increased by more than 20% (Figures 3D and 3E). Although the mouse genome cannot be directly correlated with the clinical outcome, the mouse showing the highest target indels were subsequently subjected to off-target analysis to validate their *in vivo* specificity for TS-APOC3. Among the potential off-target sites selected by the *in silico* method, none showed off-target editing (Figure 3F). This indicated that the *APOC3* editing system also specifically targeted the liver when applied with AAV-mediated delivery.

### Genome-mediated expression of F9 in humanized mouse livers

**Figure 4:**
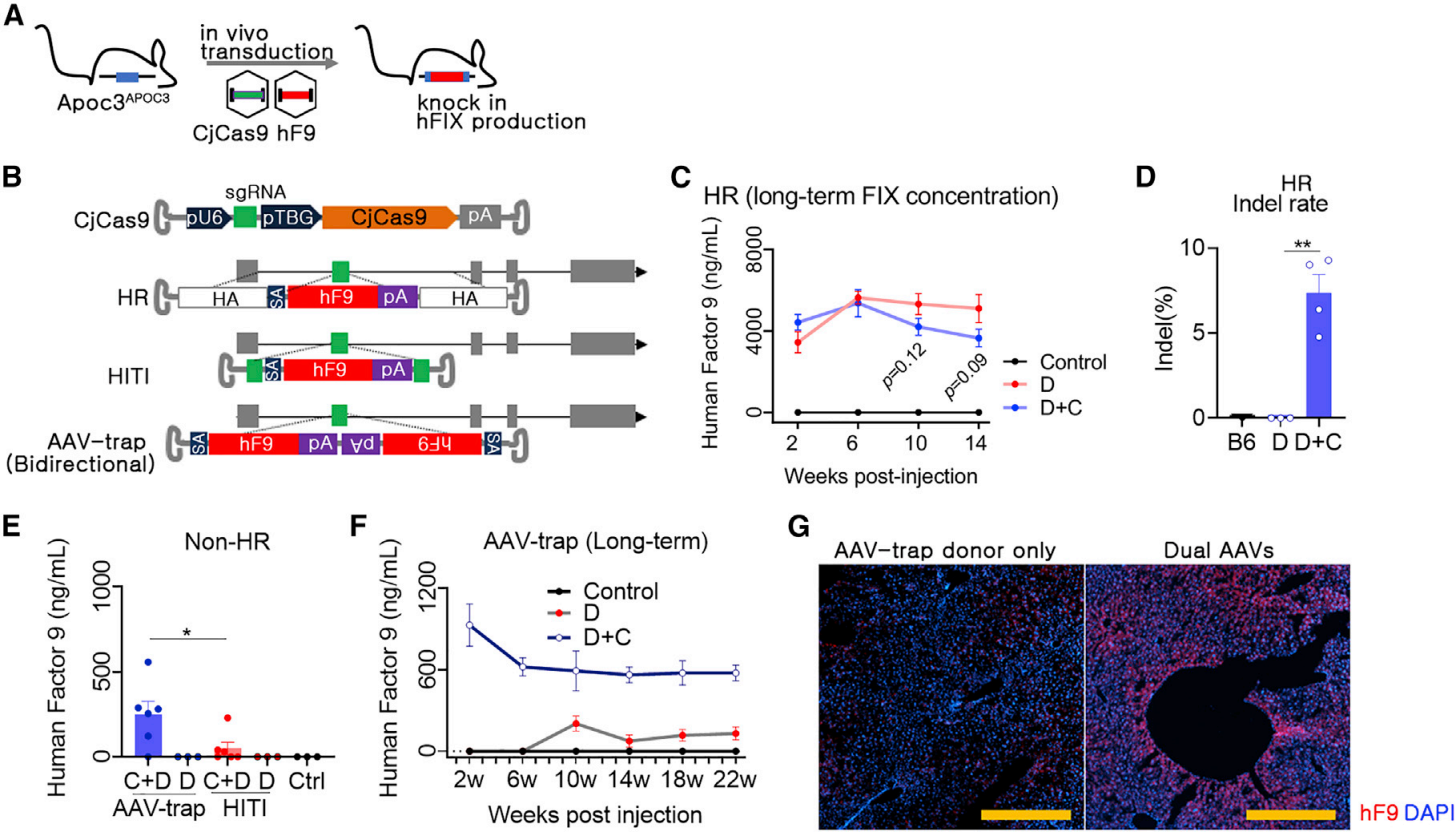
Expression of therapeutic proteins via HR, HITI, and AAV-trap approaches (A) Strategy for therapeutic gene knock-in. Dual AAVs were transduced into the humanized mice, and expression by *hF9* gene knock-in was confirmed. (B) AAV vector maps for *in vivo* KI used in this study. AAV-CjCas9 was designed with a U6 promoter, sgRNA, TBG promoter, CjCas9, and polyadenylation signal (pA). Next, three donor templates for HR, HITI, and bidirectional were designed. HA, homology arm, SA; En2SA splicing acceptor; gray box, exon. (C) HR-mediated *in vivo* expression of hFIX. Control, AAV-untreated mice (n = 4); D, AAV-HR donor only (n = 5); D + C, dual AAVs (AAV-CjCas9 + AAV-HR donor) (n = 6). AAV-HR donor only (2 × 10^13^ vg/kg) or dual AAVs (2 × 10^13^ vg/kg for each AAV) were injected intravenously. Then, blood was repeatedly sampled, and hFIX concentrations were analyzed. (D) Indel frequency at the APOC3 target site in AAV-injected liver. (E) KI-mediated expression of hFIX using a non-HR strategy was analyzed. Blood samples were collected 6 weeks after injection of 2 × 10^13^ vg/kg of each AAV. D, AAV donor only (AAV-trap or HITI); D + C, dual AAVs. Each dot indicates data from an individual mouse. Statistical analysis was performed using the Student t test. ∗p < 0.05, ∗∗∗p < 0.001, and ∗∗∗∗p < 0.0001. (F) Analysis of long-term effects by AAV-trap strategy. Control, AAV-untreated mice (n = 3); D, AAV-trap donor only (n = 3); D + C, dual AAVs (AAV-CjCas9 + AAV-trap donor) (n = 4). (G) hFIX protein expression by AAV-trap strategy was detected by immunofluorescence analysis of liver tissues. A representative image is shown.

To investigate APOC3 editing-mediated hFIX expression, we first selected the HR approach and used a dual AAV system (4 × 10^13^ vg/kg for each AAV) in humanized mice (Figures 4A and 4B). Interestingly, high expression of hFIX was observed in the HR-donor-only group, and this expression was not further enhanced by the dual AAVs. The measured hFIX level was approximately 5,000 ng/mL (Figure 4C), which was similar to that in normal human blood. Sequence analysis showed that various transcription factor binding sites and TATA/CCAAT-boxes were conserved in the upstream homology arm (HA)^29^ (Figure S6); thus, this result seemed to be due to the promoter function of the HR donor itself (Figure S6). Although this result was interesting, hFIX expression was not triggered by genome editing. Thus, we next investigated HR-independent approaches, i.e., HITI and AAV-trap, which do not use the HA. The AAV trap was designed to encode the hF9 donor bidirectionally^30^ (Figure 4B), and studies have suggested that the AAV genome can integrate into the double-strand break site.^31^ After randomly dividing the mice, the AAVs-TS-APOC3-CjCas9 and AAV donors (HITI or AAV-trap) were transduced by intravenous injection, and the hFIX concentration was measured at 6 weeks after transduction. In mice injected with AAV donors only, no hFIX production was detected for the two non-HR strategies, in contrast to the results of the HR strategy. Importantly, dual AAV-transduced groups showed evidence of hFIX production, and the plasma hFIX concentrations in the HITI and AAV-trap groups were approximately 250 and 50 ng/mL, respectively (Figure 4E). Next, we analyzed the long-term expression of hFIX in AAV-trap-treated mice, and the results showed that expression persisted for 22 weeks after AAV treatment (Figure 4F). AAV-trap-mediated hFIX expression was also verified in the immunofluorescence using anti-human FIX polyclonal antibodies of transduced hepatocytes (Figure 4G).

### Recovery of clotting function in hemophilia model mice

**Figure 5:**
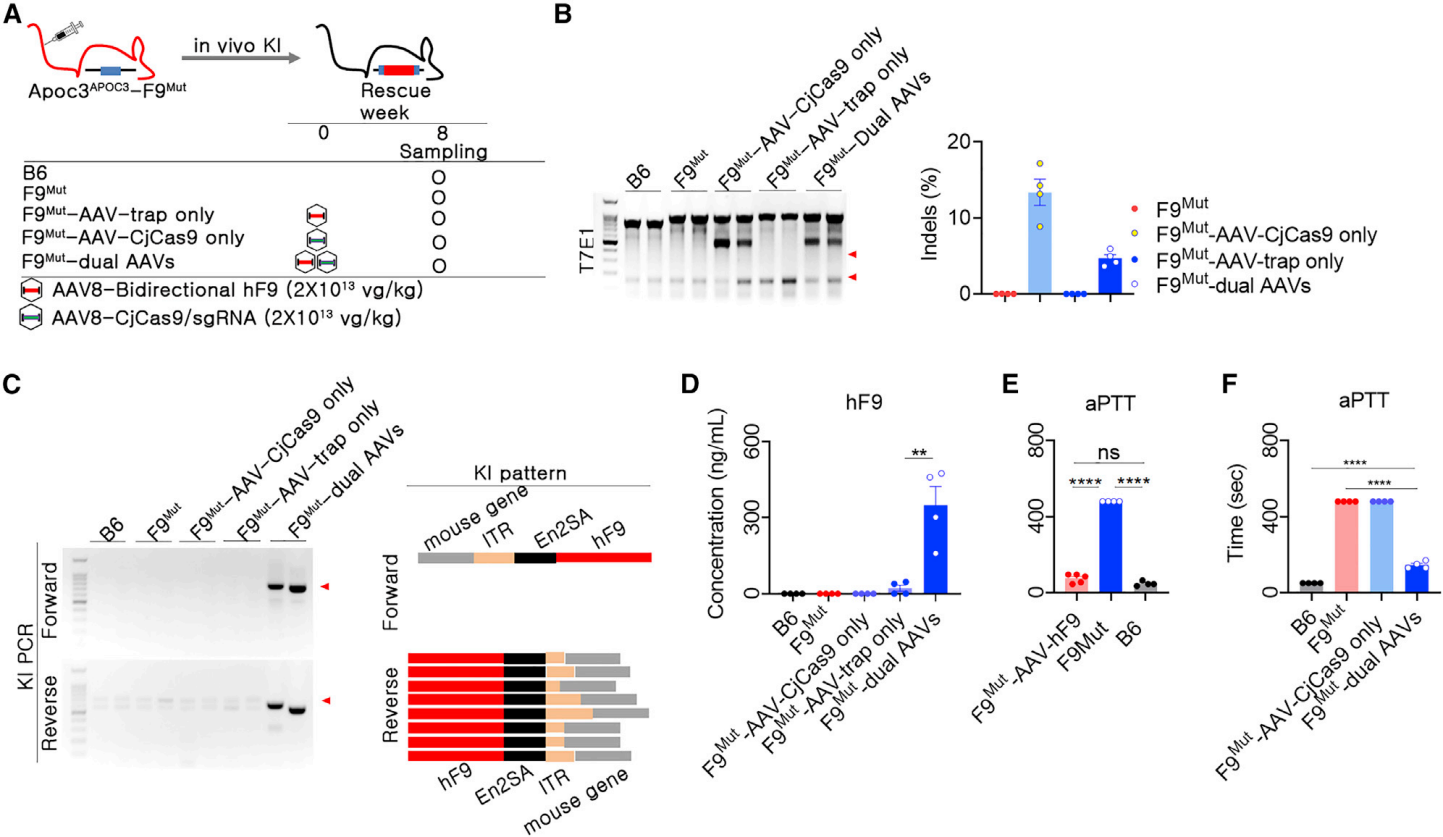
AAV-trap-mediated expression of hFIX and therapeutics effect in APOC3-humanized hemophilia model (A) Brief information for AAV transduction in dual mutant mice. (B) The indels in AAV-transduced livers were confirmed by T7E1 analysis (left) and targeted deep sequencing (right). Red arrowhead indicates DNA fragments cleaved by T7E1. (C) Confirmation of the existence of KI by PCR and analysis of KI patterns by sequencing. For KI pattern analysis, 15 clones from forward and reverse sites were subjected to sequencing and compared with the expected KI map. Yellow box, unexpected indel. (D) AAV-trap-mediated *in vivo* expression of hFIX (n = 4 for each group). (E) Cross-reactivity of hFIX and mFIX was evaluated by transduction of AAV-CMV-hF9 into F9^Mut^ mice. Plasma was collected at 6 weeks after transduction of 4 × 10^13^ vg/kg AAV into mice. The hFIX concentration was approximately 2,930 ng/mL in the AAV-CMV-hF9 group (detailed data are not shown). Next, aPTT was conducted to evaluate coagulation activity and cross-reactivity between hFIX and mFIX (n = 4 for each group). (F) *hF9* gene KI-mediated therapeutic effects were evaluated by aPTT (n = 3–4 per group). Each dot indicates data from an individual mouse. Statistical analysis was performed using the Student t test. ∗∗p < 0.01, ∗∗∗∗p < 0.0001.

To investigate whether our knock-in strategy was therapeutically applicable, we applied the AAV-trap approach in double mutant mice, which were established by crossing *APOC3*-humanized mice with F9-mutant mice (Figure 5A). As expected, *APOC3* editing was observed only when AAV delivering CjCas9 and TS-APOC3 (F9^Mut^-AAV-CjCas9) was included, as measured by T7E1 assays and targeted deep sequencing. Next-generation sequencing showed that the indel frequencies were 13.4 ± 1.7% and 4.7 ± 0.5% for single AAV (F9^Mut^-AAV-CjCas9) and dual AAVs for AAV-trap (F9^Mut^-AAV-CjCas9 + bidirectional), respectively (Figure 5B). Given that CjCas9 was expressed under liver specific promoter thyroxine-binding globulin (TBG), we also checked the biodistribution of hAPOC3 on- and off-targeting in the disease mouse treated with AAVs. The results revealed no meaningful editing at on- (Figure S7) or off-target loci (Table S3) in other tissues including lung, kidney, spleen, and heart, suggesting an evident liver-specific genomic editing. Compared with other groups, knock-in-specific polymerase chain reaction (PCR) products were specifically observed in the dual AAV-injected group (Figure 5C). Sanger sequence analysis revealed that all knocked-in sequences, including inverted terminal repeats (ITRs), splicing acceptors, and F9, were located at the precise TS-APOC3 site. Alleles with partial deletion around the ITR were observed, although the splicing acceptor and F9 sequences were relatively well preserved (Figure 5C). AAV-trap-induced hFIX expression was consistently observed in the double mutant mice (Figure 5D), and the expression was similar to that observed in humanized mice (Figure 4E). Subsequently, we investigated the effects of knock-in-driven hFIX expression on clotting function. The feasibility of the activated partial thromboplastin time (aPTT) assay to evaluate clotting function was verified using overexpression of *hF9* after injection of AAV-CMV-hF9 in the hemophilia model (Figure 5E). Importantly, abnormally increased aPTTs in the hemophilia model were significantly decreased in mice treated with AAV-trap (Figure 5F). The reduced clotting time was approximately 70% of that in normal mice, indicating substantial recovery of clotting function. Taken together, these findings suggested that the *APOC3*-targeted genome editing platform resulted in a therapeutically relevant range of hFIX expression.

## Discussion

Hemophilia B is a recessive genetic disease mainly caused by loss-of-function mutations in the *F9* gene. Although prophylaxis to supply clotting factor proteins has been developed to increase the dosing interval, the most extended long-acting treatment is still biweekly injections, and the need for frequent administration and the high cost of this therapy contribute to the low quality of life for these patients.^32^ Therefore, advanced therapeutic modalities have recently been developed to overcome these limitations.^33^ In this study, we tested the proof of concept that genome editing-mediated knock-in of *hF9* into the *APOC3* gene may have applications as an advanced therapeutic option for hemophilia B. We showed that dual AAV-mediated delivery of CjCas9 and a donor template led to the efficient expression of hFIX and recovery of clotting function in hemophilia model mice. Although this prototype platform may require additional optimization for translation, the tested platform yielded therapeutically relevant levels of hFIX expression. Moreover, the platform may have applications in various liver-targeted diseases simply by alternating the transgenes in the donor template. Importantly, we demonstrated the effectiveness of CjCas9 targeting the human *APOC3* site in a humanized mouse model and showed that the approach was highly specific in deep off-target studies, including biased and unbiased off-target detection methods, supporting the potential applications of the tested guide RNA in the clinical setting.

After demonstrating their characteristics as hepatocyte-enriched genes, *APOC3* and *HP* were selected as candidate genes whose transcriptional activity was approximately 200 times higher than those of the genes responsible for monogenic disease. Ideally, 0.5% functional knock-in of the genes in the loci with strong promoters could be sufficient to achieve a normal level of expression. In the context of hemophilia B, hFIX levels greater than 10% are needed to ameliorate disease phenotypes^34^; thus, 0.05% functional knock-in seemed to be therapeutically applicable. Accordingly, our genome editing platform could be practically feasible, despite general concerns related to low knock-in efficiency. In terms of safety, we primarily considered the effects of unwanted changes in the expression of the genes with strong promoters. Therefore, we specifically edited the middle region of the intron to avoid the risk of frameshift mutations or abnormal splicing. Moreover, even if editing resulted in expression loss, functional loss could be non-cell autonomously compensated by secretion of APOC3 or HP protein from the majority of cells because of the low editing rate. In addition, loss of function of APOC3 has been reported to be beneficial in humans.^20^, ^21^, ^22^ Accordingly, our strategy for editing *APOC3* could be an effective and safe approach.

With the application of genome editing, the knock-in of transgenes is mediated by HR or non-homologous end-joining.^5^^,^^10^ Although existing HR mechanisms have been found in post-mitotic cells,^35^, ^36^, ^37^ HR is generally effective in dividing cells. Therefore, an HR-independent strategy has been suggested to increase knock-in efficiency *in vivo*,^28^^,^^38^^,^^39^ because many cell types in the human body are terminally differentiated and do not proliferate after birth. Interestingly, when we applied the HR donor itself without AAV delivering CjCas9 and TS-APOC3, substantial expression of hFIX, close to normal levels in human blood, was observed. This highlighted the strong promoter function of the HR donor, in which the upstream HA contained a TATA box, a CAAT box, and diverse transcription binding sites. Owing to the inefficient HR rate in the adult liver combined with greater expression from the donor itself, the additive expression of hFIX by knock-in was difficult to observe when dual AAV with an HR strategy was applied. Instead, we compared two different HR-independent approaches, and knock-in-driven expression of hFIX was clearly observed for both strategies. Accordingly, the desired function of *APOC3* was verified using HR-independent approaches *in vivo*. Although the AAV trap was relatively more effective than HITI under the conditions used in our study, the effectiveness of the final outcome may differ depending on how the strategies are further optimized. However, the AAV trap method could be used flexibly for any double-strand break sites, as the donor DNA does not require any site-specific sequences, such as homology arms or sgRNA target sites that are used in HR or HITI methods, respectively. Importantly, the method with the bidirectional form could produce functional proteins regardless of their direction of integration. Interestingly, a previous study also demonstrated that a similar AAV trap method facilitated efficient production of functional proteins in mouse and non-human primates by targeting albumin gene locus with a strong promoter,^40^ suggesting it to be one of the most feasible methods for the genome editing-mediated therapy.

CjCas9, one of the smallest Cas9 proteins, is well suited for packaging Cas9 and sgRNA with corresponding promoters in a single AAV. Accordingly, the effective *in vivo* application of CjCas9 using a single AAV has also been reported in a model of wet age-related macular degeneration.^23^ In addition to efficient packaging in AAV, relatively higher specificity compared with SpCas9 was also demonstrated by direct comparison using a target site having both NNNNRYAC and NGG PAM.^23^ Such remarkable specificity of CjCas9 was verified in the mouse retina at 18 months after AAV treatment.^41^ We also performed off-target studies using biased and unbiased methods, and no active off-target loci were detected in human cells or AAV-treated livers, indicating the high specificity of our CjCas9 targeting *APOC3* or *HP* genes. The combination of biased and unbiased off-target detection methods has been recommended by the U.S. Food and Drug Administration guidelines, and a similar approach was proposed in Investigational New Drug applications for recently entered clinical trials targeting blindness and inherited liver disease.^6^^,^^42^ The advantage of the specificity of CjCas9 could be critical in AAV application, which generally expresses Cas9 over a long-term period, thereby increasing the risk of off-target loci. Alternatively, nonviral delivery of Cas9 together with an AAV donor could decrease the off-target risk, as reported in a previous study of correction of liver enzymes.^43^

The systemic administration of high-dose AAVs has recently been shown to be associated with severe liver- or immune-related toxicities in clinical studies of X-linked myotubular myopathy and Duchenne muscular dystrophy.^44^^,^^45^ Similarly, severe liver toxicity has been reported in non-human primates (NHPs) intravenously treated with high doses of AAVs.^46^ The doses used to induce toxicities were greater than 10^14^ vg/kg, which may be the highest AAV dose that is safe for clinical application. Although we observed increased delivery of AAV copies and editing in high-dose-treated mice (1 × 10^14^ vg/kg), we used a middle dose (4 × 10^13^ vg/kg) to measure therapeutic expression or recovery of the disease phenotype because of the potential for toxicity. These results could be critical because the safe dose range resulted in therapeutic outcomes, possibly because of *APOC3* activity. However, the low editing efficiency at the middle dose could still be unsatisfactory or may not be promising in NHPs or humans; accordingly, further optimization of our vector is required. Although optimization of our platform is beyond the scope of the current study, various factors could be considered for the improvement of CjCas9 expression because its small size will allow for the incorporation of additional elements, even when considering the packaging of AAV. These factors include promoter-compatible enhancer motifs, codon usage, poly-As, and untranslated regions, such as the woodchuck hepatitis virus post-transcriptional response element.^47^ In addition, engineered AAV capsids with efficient liver entry and transduction^48^ may facilitate therapeutic application at a safe dose range.

In conclusion, in this study, we established a useful CRISPR/Cas9-based platform to treat hemophilia. Our genome editing approach could provide a versatile option for solving the unmet need for long-term therapeutic effects in patients with genetic liver diseases and may therefore complement other advanced therapies, including AAV-mediated gene replacement therapies.

## Materials and methods

### Cell culture and transfection

HEK293 cells (American Type Culture Collection [ATCC], Manassas, VA; cat. no. CRL-1573) and Hep G2 cells (ATCC; cat. no. HB-8065) were maintained in Dulbecco’s modified Eagle’s medium and Roswell Park Memorial Institute, respectively, supplemented with glucose (4.5 g/L), 4 mM glutamine, 1 mM sodium pyruvate, 10% fetal bovine serum, penicillin (100 U/mL), and streptomycin (100 mg/mL). Hep G2 cells were transfected with plasmids using Neon Transfection (Life Technologies, Carlsbad, CA) according to the manufacturer’s instructions. Briefly, 1.5 × 10^5^ cells were resuspended to 10 μL of R buffer in Neon Transfection kit. Subsequently, 1230 V/20 ms/3 pulses Neon system is used to electroporate the cells. Two days after transfection, the cells were harvested, and genomic DNA was extracted using a FavorPrep blood/cultured cell genomic DNA extraction mini kit (FAVORGEN). For transfection of HEK293 cells, 5 × 10^4^ cells were seeded the day before transfection in 24-well plates. The next day, Total 1 μg plasmids encoding human optimized F9 donor and CjCas9 was diluted to 200 μL Opti-MEM and mixed with diluted lipofectamine 2000 (1:2 ratio) and then incubated for 20 min and added to cells. Genomic DNA was isolated after 4 days of transfection.

### RNA-sequencing

Human primary hepatocytes purchased from ThermoFisher Scientific (Waltham, MA) were maintained in medium containing supplements for 72 h and subjected to RNA purification using a purification kit. After checking the purity of the RNAs (RNA integrity number >7, 28s/18s 1–3) using an Agilent 2100 bioanalyzer (Agilent Technologies, CA; a cDNA library was constructed using a TruSeq RNA Library Prep Kit (Illumina, San Diego, CA). The final library sizes and qualities were evaluated electrophoretically using an Agilent High Sensitivity DNA kit (Agilent Technologies), and the library was subsequently sequenced using an Illumina HiSeq2500 sequencer (Illumina). For transcriptome data analysis, filtered sequence reads were mapped to the human reference genome (Ensembl release 72) using the aligner Tophat. Gene expression levels were measured with Cufflinks v2.1.1, using the gene annotation database of Ensembl release 72. The study of human hepatocytes was approved by the Institutional Review Board of Toolgen (approval no. TG-human-2021001-P).

### sgRNA screening and targeted deep sequencing

For the sgRNA activity screening, plasmids delivering EFS-CjCas9 and U6-sgRNA for each target sites were constructed. Upon transfection of the plasmids into human cell line HEK293, recombinant plasmids were compared for indel frequency. Target sites were initially identified as 22 nucleotides adjacent to the PAM NNNNRYAC in the first intron of human HP or APOC3 gene using *in silico* tool, Cas9 Designer (www.rgenome.net). Among them, target sites having 1- or 2-bp mismatched genomic sites were considered as low specific sites and excluded from the activity screening assay owing to safety concerns.

Precise quantification of on-target and off-target editing was performed by targeted deep sequencing. The sequences of the tested on-target or off-target sites are listed in Table S1. Briefly, on-target and off-target regions were amplified from genomic DNA (100 ng) extracted from transfected cells or AAV-treated livers using Phusion High-Fidelity DNA Polymerase (New England Biolabs, Ipswich, MA) with specific primers (Table S4). The PCR products were further amplified using Illumina TrueSeq adaptors and purified using fragment DNA purification kit (Intronbio, Houston, TX). After pooling in same molar ratio, the libraries were sequenced in both directions using Miseq with a TrueSeq HT dual index system (Illumina). The indel frequencies were quantified using a Cas-Analyzer (www.rgenome.net). Indels in the region 3 bp upstream of the PAM sequence NNNNRYAC were considered mutations resulting from CjCas9.

### Off-target study: Digenome-seq

To detect genome-wide potential off-targets, Digenome-seq^49^ was performed. Briefly, human genomic DNA (8 μg) was cleaved with CjCas9 (300 nM) and sgRNA (900 nM) in total 400 μL reaction volume (100 mM NaCl, 50 mM Tris-HCl, 10 mM MgCl_2_, and 100 μg/mL BSA) *in vitro*. and then, incubated for 8 h at 37°C. Digested genomic DNA was treated with RNase A (50 μg/mL) for 30 min at 37°C to remove sgRNAs and purified again using a DNeasy Tissue kit (Qiagen, Valencia, CA). The purified DNA was fragmented using the Covaris system (Life Technologies) to obtain a product of 300–400 bp average size and blunt-ended using End Repair Mix (ThermoFisher Scientific). The final DNA library was generated by ligating with adaptors to the end of the DNA fragments and then sequenced by whole-genome sequencing using an Illumina HiSeq X Ten Sequencer. After mapping the sequenced reads to the human genome reference hg19 using Isaac aligner, the produced Bam files were analyzed using Digenome-sequencing tools (www.rgenome.net) to obtain off-target loci. We selected the loci showing cleavage scores greater than 0.1 as potential off-target sites (Table S1) and further verified these loci by targeted deep sequencing.

### Flow cytometry

Hep G2 cells were transfected with GFP HITI donor plasmid in the absence or presence of the plasmid delivering CjCas9 and TS-APOC3. The cells were harvested at multiple time points from 3 days to 3 weeks after transfection and then subjected to GFP expression analysis using fluorescence-assisted cell sorting (FACS) using an Attune Life Technologies instrument according to the manufacturer’s instructions. Briefly, after resuspending the cell pellets in 200 μL FACS buffer (1× phosphate-buffered saline [PBS] supplemented with 2% fetal bovine serum), the cells were gated by forward and side scatter and were subsequently analyzed in density plots displaying GFP fluorescence in the green channel. Ratios of the number of enhanced green fluorescent protein (EGFP)-positive cells among a total of 20,000 cells were quantified and compared at the indicated time points. Hep G2 cells treated with pAAV-CMV-EGFP were used as controls.

### Preparation of sgRNA and ssODN

The sgRNA targeting mouse *Apoc3* intron 1 were designed with a 20-bp binding sequence with the PAM sequence (5′-NGG-3′) of *Streptococcus pyogenes* (SpCas9). The sequences were synthesized using an *in vitro* RNA synthesis kit (ThermoFisher Scientific) after PCR amplification. The ssODNs were designed with a total of 200 bp, including 40 bp HAs on both sides and a 120-bp human *APOC3* sequence in the middle (HA-hAPOC3 sequence-HA). ssODNs were prepared using a commercial service (Integrated DNA Technologies, Skokie, IL). The detailed sequences of the target site and ssODNs are listed in Table S2.

### Generation of human sequence knock-in mice

C57BL/6 (B6) mice were purchased from Koatech (Pyeongtaek, Korea), and embryos were collected after superovulation. Next, the embryos were subjected to gene editing by electroporation. Briefly, 200 ng/μL SpCas9 protein (Toolgen Inc., Seoul, Korea), 50 ng/μL sgRNA, and 100 ng/μL ssODN were transfected into embryos by electroporation.^50^ Then, embryos were transferred to the oviducts of surrogate ICR female mice, and founder mice were confirmed by PCR genotyping and Sanger sequencing. The study was approved by the Institutional Animal Care and Use Committee of Seoul National University (approval nos. SNU-170816-5, SNU-170816-6, and SNU-2007715-2).

### Quantitative reverse transcription PCR

Total RNA was extracted from liver tissues, and cDNA was synthesized. Next, gene expression was compared by quantitative reverse transcription (RT)-PCR for *F9*, alpha-L-iduronidas, iduronate 2-sulfatase, galactosidase-alpha, *GBA*, albumin, and *Apoc3*. Quantitative RT-PCR was conducted in quadruplicate with primers obtained from the Primer Bank (https://pga.mgh.harvard.edu/primerbank/) using SYBR premix (ThermoFisher Scientific), and gene expression levels were normalized to that of *GAPDH*. The sequences of the primers used are listed in Table S5. The AAV genome copy number in liver samples was determined using an AAVpro titration kit (Takara, Shiga, Japan) according to the manufacturer’s instructions. The number of AAV genome copies was determined against a standard curve. A conversion factor of ∼1.6 × 10^2^ diploid cells per 1 μg mouse genomic DNA was used to calculate the number of diploid cells.

### Recombinant AAV preparation

To prepare recombinant AAV vectors for AAV2-CMV-eGFP-pA (AAV-eGFP), AAV2-TBG-CjCas9-pA-U6-sgRNA (AAV-CjCas9), AAV2-CMV-eGFP-pA (pAAV-eGFP), AAV2-HA-En2SA-hF9-pA-HA (AAV-HR), AAV2-sgRNA-En2SA-hF9-pA-sgRNA (AAV-HITI), and AAV2-En2SA-hF9-pA-pA-hF9-En2SA (AAV-bidirectional) were prepared by DNA synthesis. To synthesize each AAV, helper and serotype-specific Rep/cap plasmids were cotransfected into HEK293 cells, and the viral particles were collected after 3 days. AAV-eGFP was produced by AAV serotypes 8 and 9, whereas all other AAVs were prepared as AAV8 serotypes. After purification and titration by quantitative RT-PCR, the AAVs were stored at −80°C until use. The recombinant AAVs (rAAVs) were prepared using Vigene Biosciences (Rockville, MD).

### *In vivo* rAAV transduction

To select the candidate AAV serotype, AAV8-eGFP or AAV9-eGFP were transduced into 8-week-old B6 mice by intravenous injection, and liver tissue was subjected to further detection of GFP signals after immunofluorescence. To evaluate the *in vivo* knock-in efficiency of AAV, 8-week-old mice were used. AAV8-CjCas9 or AAV8-donor (AAV-HR, AAV-HITI, or AAV-bidirectional) particles were mixed with warm saline to a volume of 200 μL and injected intravenously or intraperitoneally. When dual AAV8-CjCas9 and AAV8-donor AAVs were transduced, 400 μL of the AAV mixture was injected into a single mouse. Next, hemophilia B mice with *F9* knockout (F9^Mut^) were used to assess the therapeutic effects of *hF9* gene knock-in. F9^Mut^ mice were monitored weekly for abnormal appearance and behavior after AAV transduction. Viral titers and combinations are described in the relevant figure legends.

### hF9 ELISA

Plasma was collected from the tail vein or inferior vena cava using heparinized tubes and was then centrifuged. Blood hFIX concentrations were measured with 200-fold dilution using a Factor 9 Human Simple Step ELISA kit (Abcam, Cambridge, UK) according to the manufacturer’s instructions. A value of less than 0 was designated as undetected.

### Immunofluorescence

Liver tissues were cryo-fixed after perfusion using prewarmed PBS, fixation in 4% paraformaldehyde, and precipitation in 20% sucrose. Slides were then blocked with control serum, incubated with primary rabbit anti-human FIX polyclonal antibodies (Catalog #ab97619, 1:200 dilution, Abcam) at 4°C, overnight and incubated again with Goat anti-rabbit Alexa Fluor 594 secondary antibody (Catalog #A11012, 1:400 dilution, ThermoFisher Scientific) at room temperature for 1 h. DAPI were used to stain nucleus. The fluorescent signal was detected using a Cytation 5 instrument (BioTek, Winooski, VT).

### aPTT analysis

Blood (450 μL) was collected with 50 μL of a 3.2% sodium citrate solution (Medicago, Durham, NC) from the inferior vena cava, and plasma was collected after centrifugation. In microplate-based aPTT analysis, 30 μL plasma and aPTT reagent (ThermoFisher Scientific) were mixed in 96-well microplates and incubated at 37°C for 5 min. Next, 30 μL 26 μM CaCl_2_ was added to the incubated serum-reagent mixture. Absorbance was measured every 10 s for 8 min at 405 nm with shaking. The time point with the highest ΔOD(Time[n+1], Time[n]) value was selected as the result value of the aPTT test.

### Statistical analysis

Statistical analysis was performed using unpaired Student t tests with GraphPad Prism (version 5.02; GraphPad, San Diego, CA).

## Data Availability

All data described in this study are available from the corresponding author upon reasonable request.

## Supplemental information

Document S1. Figures S1–S7 and Tables S1–S5

Document S2. Article plus supplemental information
