# Supplementary material for: Genome editing-mediated knock-in of therapeutic genes ameliorates the disease phenotype in a model of hemophilia
Source: Mol Ther Nucleic Acids. 2022 Aug 4;29:551–62. doi: 10.1016/j.omtn.2022.08.002 (PMC9403902; doi:10.1016/j.omtn.2022.08.002)
Supplement: Document S1. Figures S1–S7 and Tables S1–S5 [file mmc1.pdf]

OMTN, Volume *29*

Supplemental information

Genome editing-mediated knock-in of therapeutic genes ameliorates the disease phenotype in a model of hemophilia

Jeong Hyeon Lee, Hye-Kyung Oh, Beom Seok Choi, Ho Hyeon Lee, Kyu Jun Lee, Un Gi Kim, Jina Lee, Hyerim Lee, Geon Seong Lee, Se Jun Ahn, Jeong Pil Han, Seokjoong Kim, Su Cheong Yeom, and Dong Woo Song

Supplementary figures

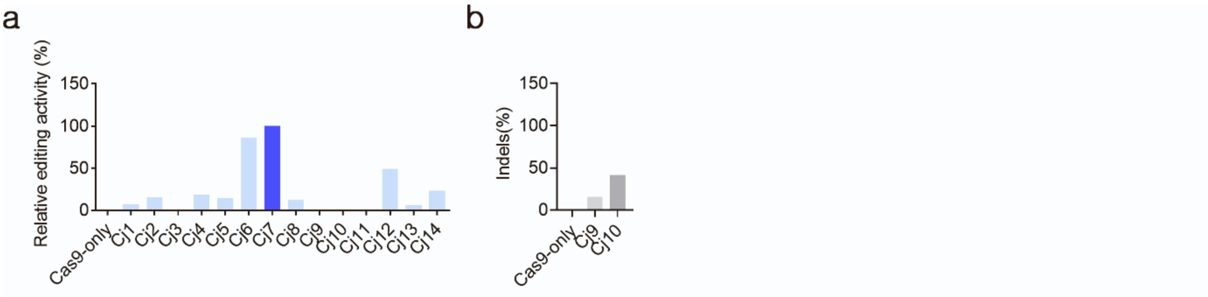

Figure S1. Screening of sgRNAs targeting the *APOC3* or *HP* gene in human cells. (a, b) Indel frequencies induced by the tested sgRNAs targeting intron 1 of *APOC3* **(a)** and *HP1* **(b)**. In screening for the *HP* gene, 10 sgRNAs were initially tested, and two sgRNAs effective in T7E1 assays (data not shown) were further analyzed by targeted deep sequencing **(b)**.

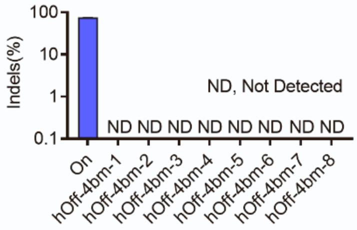

Figure S2. Off-target analysis of in silico-based candidates of TS-APOC3. Targeted deep sequencing results for the eight most homologous sites that differed by four nucleotides from the TS-APOC3 site. 4bm, 4 base mismatches. The indel frequency was measured in HEK293 cells, and the on-target indel frequency was 76.6% ± 0.9%.

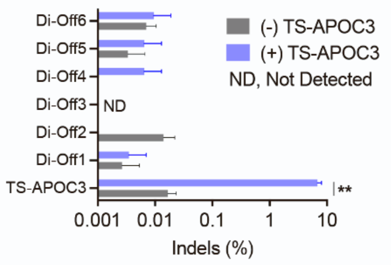

Figure S3. Off-target analysis of TS-APOC3 in hepatocyte-like cells. Targeted deep sequencing results for the off-target candidates obtained by Digenome-seq in TS-APOC3-treated Hep G2 cells. (n = 3). Results presented are the means ± standard errors obtained from three independent experiments; statistical analysis was performed using Student's t-test. ***p* < 0.01

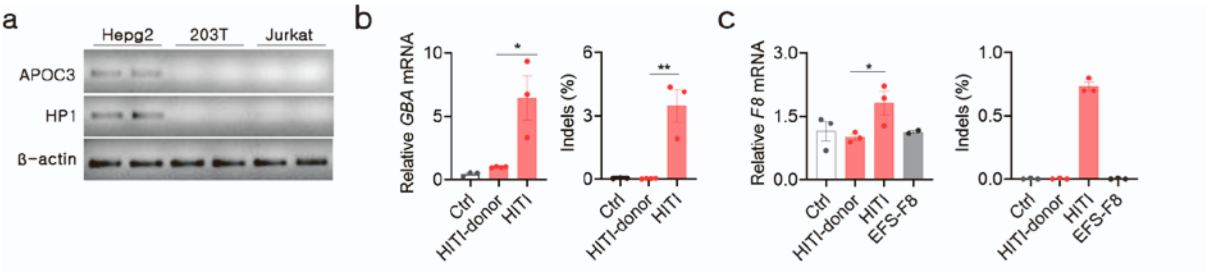

Figure S4. Expression of therapeutic proteins in Hep G2 cells. (a) RT-PCR results showing *APOC3* mRNA expression in Hep G2 cells. **(b)** *GBA* mRNA expression (left panel) and indel frequency (right panel) in HepG2 cells transfected with HITI donor in the absence or presence of the plasmid expressing CjCas9 and TS-APOC3 (n = 3). **(c)** *F8* mRNA expression (left panel) and indel frequency (right panel) using the same strategy (n = 3). Ctrl: untransfected cells. Statistical analysis was performed using Student's t-test. *$p < 0.05$.

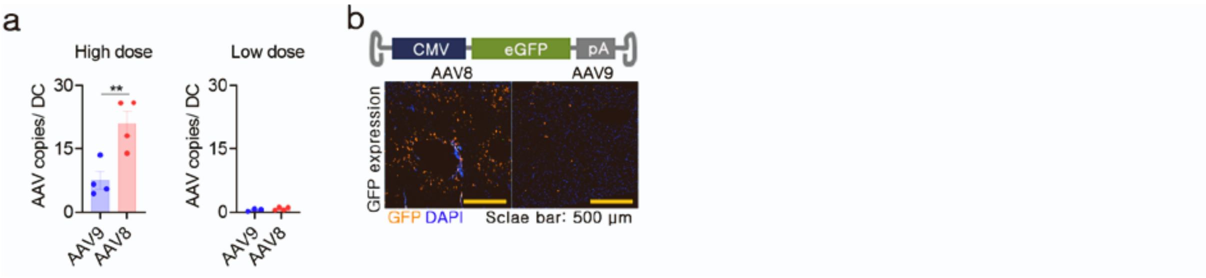

Figure S5. Comparison of AAV serotypes for in vivo delivery. (a) Quantification of AAV genome copy numbers per diploid cell in livers 6 weeks after injection of AAV8 and AAV9 delivering CjCas9 and sgRNA targeting mROSA (n = 3–4). Low dose, 1×10^{12} vg/kg; High dose, 1×10^{13} vg/kg. **(b)** Immunofluorescence images from liver sections 3 weeks after injection of AAV8 and AAV9 delivering CMV-EGFP. 1×10^{12} vg/kg AAVs was transduced via the tail vein. Statistical analysis was performed using Student's t-test. $^{**}p < 0.01$.

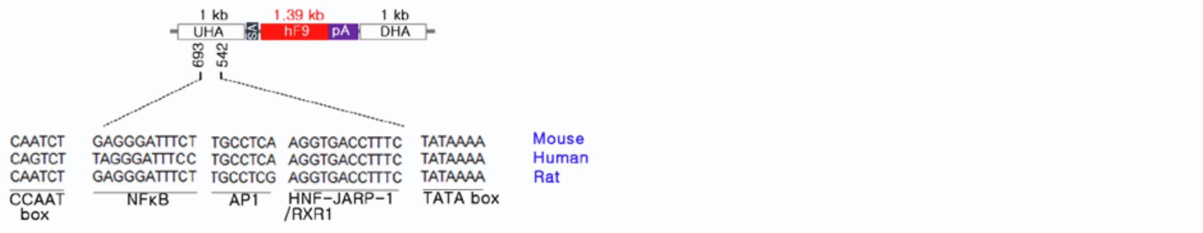

Figure S6. Promoter sequences of the upstream homology arm of the HR donor for TS-APOC3. Schematic drawing of the HR donor for TS-APOC3 and potential sequences for the transcription of hF9. TATA box, CCAAT-box, and some transcription binding sites were located in the UHR, and the potentially critical sequences were well conserved among humans and rodents. UHR, upstream homology arm; DHR, downstream homology arm; SA, splicing acceptor; pA, bovine growth hormone polyadenylation signal. The numbers displayed below the UHR indicate the location from the start of the homology arm.

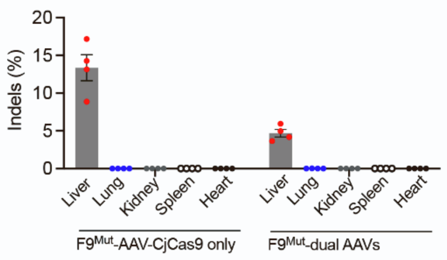

Figure S7. Biodistribution of on-target editing in the diseased mouse treated with AAVs. Targeted deep sequencing results for the TS-APOC3 on-target editing on the loci of liver, lung, kidney, spleen, and heart of the F9^{Mut} mouse treated with AAV-CjCas9 only or dual AAVs for AAV-trap (n = 4).

Supplementary tables

Table S1. On-target or Off-target sites tested in this study.

Red, mismatched nucleotides to the corresponding on-target sites; Blue, PAM sequences

Target sites	Target sequence with PAM (5'-3')
hAPOC3 On-target (TS-APOC3)	GAGAGGGCCAGAAATCACCCAAAGACACAC
hAPOC3-di-off 1	GAGAGGGCAGGAAATCACCCAAGGTCACAC
hAPOC3-di-off 2	GAAGGGTTTAGAAATCACCCAAATCCACAT
hAPOC3-di-off 3	TGAAGAAAGAGAAATCACCCAATTTCACAT
hAPOC3-di-off 4	TAGCCTAATAGAAATCACCCAAATTGACAC
hAPOC3-di-off 5	AGAATTTAAAGAAATCACCCAAGAGCACAC
hAPOC3-di-off 6	AGGATCAGCAGAAATCACCCAACCTAGTAC
hHP1 On-target (TS-HP)	CATTCAGGAAAGTACATTGGCAATAATAC
hHP1-di-off 1	AGGCAGGAAAAATACATTGGCAAAAACAT
hHP1-di-off 2	ATTTGCGGAAAG-ACATTGGCTTTAGTAC
hHP1-di-off 3	CTAGATTAAATGTACATTGGCCAAAACAT
hHP1-di-off 4	CACTCCAGAAAGTAC-TTGGCTGCCACAC
hHP1-di-off 5	TGCTATTGTAAATACATTGGCCTGTACAC
hHP1-di-off 6	TTTGGGGGAAAGTACA-TGGCATAAACAC
hHP1-di-off 7	TGTTGAAGAATGTACATTGTCTCACACAC
hHP1-di-off 8	TGTTGAAGAATGTACATTGTCGCACACAC
hHP1-di-off 9	TTGGGGAGAAAG-ACATTGGCATTCACAC
hHP1-di-off 10	AGAGAAAGAGAGTACATTGGCGAAGATAC
hAPOC3-off 1	GAGAGGTTAAGAAACCACCCAATGTCGCAC
hAPOC3-off 2	GAGAGGTACAGAAATCACACACAAACATAC
hAPOC3-off 3	CAGGGGCCCAGACATCACCCAACTTTGTAC
hAPOC3-off 4	GAGAGGGCCAGACTTTGCCCAAGGTCACAC
hAPOC3-off 5	GTAAGGGCCAGAATTAACCCAAAGAAACAC
hAPOC3-off 6	GAGGGGGCAGGAAACCACCCAAGAGCACAC
hAPOC3-off 7	GAGAGGGTAAGAAACCACCCATGACCACAC
hAPOC3-off 8	GAGAGGGCAGGAATTTACCCAAGGTCACAC
mAPOC3-off 1	GGAAAGGCCAGAAATCACtCAAGAAAGTAC
mAPOC3-off 2	GAGAGGGTCATACATTACCCAAGGCCACAC
mAPOC3-off 3	AAGATGGCCAGAATTCACTCAAGGGGACAC

mAPOC3-off 4	GAGAGGCTAAGAAATCACCTAAGGTCACAC
mAPOC3-off 5	GAGAGGCCCAGGAATTACCCACCCGGGTAC
hAPOC3-Cj1	CCAGCCCAGCCAGCAAGCCTGGAGAAGCAC
hAPOC3-Cj2	CTTCAGGTTATGATGAGGGGTGGGGGGCAC
hAPOC3-Cj3	GGGAGGGGTGTCACTTGCCCAAAGCTACAC
hAPOC3-Cj4	ACCCCCTGTGTAGCTTTGGGCAAGTGACAC
hAPOC3-Cj5	AAGCCTGAAGAATGAGGGGGGAACCTGCAC
hAPOC3-Cj6	TGGAGAGGGCCAGAAATCACCCAAAGACAC
hAPOC3-Cj7	GAGAGGGCCAGAAATCACCCAAAGACACAC
hAPOC3-Cj8	GAAAACCCACCAGACTGAACATCAAGGCAC
hAPOC3-Cj9	AAGGAGTAGGGGCCGGCTCCCTGCTAATAC
hAPOC3-Cj10	TGGGGACCTGGGGTGCCCCTCACAGGACAC
hAPOC3-Cj11	TCCTGCAAGGAAGTGTCCTGTGAGGGGCAC
hAPOC3-Cj12	GGAACAGAGGTGCCATGCAGCCCCGGGTAC
hAPOC3-Cj13	CAACAAGGAGTACCCGGGGCTGCATGGCAC
hAPOC3-Cj14	GAGCGCCAGGAGGGCAACAACAAGGAGTAC
hHP-Cj9	TCCAGGAAAGAGAAACCTCCCTCAGACAC
hHP-Cj10	CATTCAGGAAAGTACATTGGCAATAATAC

Table S2. Sequences for the mouse APOC3 target sites and ssODN used in generation of the humanized mouse

Red, PAM sequence of SpCas9 (NGG) or CjCas9 (NNNNRYAC); Blue, homology arm sequence

Mouse APOC3 target site	ACCCAGCCCCCCAAGTGGGGTGG<br>AGCCCCCCAAGTGGGGTGGAAGG
ssODN(5' - 3')	GTCCCGTTCTGCAGAGTATTTCTATACTCCACCTTCCACCCAGGTTCCCCCCTCATTCTTCAGGCTTAGGGCTGGAGGAAGCCTTAGACAGCCCAGTCCTACCCCAGACAGGGAAACTGAGGCCTGGAGAGGGCCAGAAATCACCCAAAGACACACAGCACCACTTGGGGGGCTGGGTCTACTGTAGTTCTCTATCTAAT

Table S3. Biodistribution for on- and off-targeting genes in disease mouse treated with AAVs

Group	Tissue	Target	Mean Indels (%)	STDEV (%)
F9^{Mut}-AAV-CjCas9 only	Liver	On	13.36	2.99
		mOff-1	0	0
		mOff-2	0.01	0.01
		mOff-3	0	0
		mOff-4	0.01	0
		mOff-5	0.01	0
	Lung	On	0	0
		mOff-1	0	0
		mOff-2	0.01	0
		mOff-3	0	0
		mOff-4	0.01	0
		mOff-5	0.01	0
	Kidney	On	0.01	0.01
		mOff-1	0	0
		mOff-2	0.02	0.01
		mOff-3	0	0
		mOff-4	0.01	0
		mOff-5	0.01	0
	Spleen	On	0.01	0
		mOff-1	0	0
		mOff-2	0.02	0
		mOff-3	0	0
		mOff-4	0.01	0
		mOff-5	0.01	0.01
	Heart	On	0	0
		mOff-1	0	0
		mOff-2	0.01	0
		mOff-3	0	0
		mOff-4	0.01	0
		mOff-5	0.01	0.01
F9^{Mut}-dual AAVs	Liver	On	4.69	0.86
		mOff-1	0	0
		mOff-2	0.01	0.01
		mOff-3	0	0.01
		mOff-4	0.01	0.01

		mOff-5	0.02	0.01
	Lung	On	0.02	0.01
		mOff-1	0	0
		mOff-2	0.02	0.01
		mOff-3	0	0
		mOff-4	0.01	0.01
		mOff-5	0.02	0.01
	Kidney	On	0.01	0.01
		mOff-1	0	0
		mOff-2	0.01	0.01
		mOff-3	0	0
		mOff-4	0.01	0.01
		mOff-5	0.02	0.01
	Spleen	On	0.01	0
		mOff-1	0	0
		mOff-2	0.01	0
		mOff-3	0	0
		mOff-4	0.01	0.01
		mOff-5	0.02	0.01
	Heart	On	0.01	0
		mOff-1	0	0
		mOff-2	0.01	0.01
		mOff-3	0	0
		mOff-4	0.01	0
		mOff-5	0.02	0.01

Table S4. Primer sequences used in targeted deep-sequencing

**Target sites**	**Primer Sequence (5'-3')**	**Product size**
hAPOC3 on-target	F: AAGTGGCTCCAAGTGCAG<br>R: ATCGAGGCCCAAAGGGAG	238bp
hAPOC3 di-off-target 1	F: AGGGTTAGGTGAGATGG<br>R: AGGGAGCTACTGAAGAAC	235bp
hAPOC3 di-off-target 2	F: ACAATGAGCTTTGTGGTG<br>R: AGAGTGAGACTCTTTCTC	258bp
hAPOC3 di-off-target 3	F: AGCTACATTTTACACCTTC<br>R: TTTTCCAAGGCAGATGTG	212bp
hAPOC3 di-off-target 4	F: TGGACTTTTGCACGACTG<br>R: ACAGTCAAATGGAAGTAC	246bp
hAPOC3 di-off-target 5	F: ATGAATGTTCGGAGTAAC<br>R: ATGCACCCATTATTGTGC	269bp
hAPOC3 di-off-target 6	F: TAACCGAGATGCTCAGAG<br>R: TGGAGTCAGGAGGTACG	247bp
hHP1 On-target	F: TATCAAGAAGTAGAGGAATAGC<br>R: TCCTGCTGGGAATTCTG	227bp
hHP1 di-off-target 1	F: TTGTGTAACGTGCTGTAC<br>R: TTAGTGGAGAACTTGCTG	263bp
hHP1 di-off-target 2	F: TCCTACAGTTGAAATTGC<br>R: ACCACTACAGTCTGGAAC	242bp
hHP1 di-off-target 3	F: TGTGCATGTGTTACACTC<br>R: AATTCAGCAGACATGATG	259bp
hHP1 di-off-target 4	F: TTAGCACTTAGCACCATG<br>R: TCAGACCAGTTGAGACTG	231bp
hHP1 di-off-target 5	F: ATCAGAGGTCTGTCATTC<br>R: TTTCCAAATTGCCTCATC	189bp
hHP1 di-off-target 6	F: TCTGTTCACTGCTGCTAG<br>R: ATCCCAGCACTTTGAGAG	254bp
hHP1 di-off-target 7	F: AGTGCTGGAATTAGAGGTG<br>R: AGACAACAGCAATGGATG	238bp
hHP1 di-off-target 8	F: AAGTGATCCTCTCACTTC<br>R: ACCAAGAGAATCGAACTG	282bp
hHP1 di-off-target 9	F: ACTTTCCATGGACAAAGTC<br>R: TGACATTCCGAACTGATG	231bp

hHP1 di-off-target 10	F: TCCTTCAAGGAGTTCGTG<br>R: TCTCCAACTATGATTGTGC	267bp
hAPOC3 off-target 1	F: GCTAGAGTGCCTGGATTTGG<br>R: TCTTACAATCTGCCAAATGACC	201bp
hAPOC3 off-target 2	F: AACACCTGTCACACACACTTCA<br>R: CTCAGGGAAGACCCAGGAGT	281bp
hAPOC3 off-target 3	F: GACAGTGGAACCCCTTTCCT<br>R: CCTGAGGCTACCACTTGGAT	277bp
hAPOC3 off-target 4	F: ATCTCTGGTTGGAGGCATTG<br>R: AGGCAGGGATTCAGGAAACT	249bp
hAPOC3 off-target 5	F: GGCTTTCCCTATGCACAAAA<br>R: TGCAAGGACGATGTCAAAAG	250bp
hAPOC3 off-target 6	F: TGGGAGAAAGGGATCTGATG<br>R:<br>TCCCTTTGCTGTTTAGATAGCTG	291bp
hAPOC3 off-target 7	F: GGAATGTCCTGGAACAGTGG<br>R: TTGTCTTGCTGCTTTCATGG	265bp
hAPOC3 off-target 8	F: AAGCTGTTTCCACAGCACCT<br>R: TCAAGTCCAACTGCATCCAC	207bp
mAPOC3 off-target 1	F: GGTATGGGGGACTTTTGGTA<br>R: AACCACATGCCTCTGTCTCC	251bp
mAPOC3 off-target 2	F: AGGTTAAGTCAGGGCGGTCT<br>R: TGTGTACCACGTGCCATTTT	248bp
mAPOC3 off-target 3	F: CTGGTCCATCCTCCATCTGT<br>R: CCAGCCTTGAAGATTGATGC	200bp
mAPOC3 off-target 4	F: CCCTGCTATCCCAGGAGAC<br>R: CGAATCGCTGCACAATTTTA	202bp
mAPOC3 off-target 5	F: CGTTGTCAGTCCAGGAGGAT<br>R: CATTGACAGTGACCCCACAC	240bp

Table S5. Primer sequences used in q-RT PCR

**Target gene**	**Primer Sequence (5'-3')**	**Product size**	**Primer bank ID**
hGAPDH	F: GTCTCCTCTGACTTCAACAGCG<br>R: ACCACCCTGTTGCTGTAGCCAA	131bp	
hF9-CDS	F: CGAGAACGCCAACAAGATCCT<br>R: AGCTGCACTTCTCTTCCATGC	110bp	
hAPOC3-hF9-KI specific	F: CCTGCCTGTCTGCTCAGTTC<br>R: CTCGTGGTCCAGAAACACGG	148bp	
hF8 BDD	F: GGGAGTCTGGCCAAGGAAAA<br>R: ATGCAGCATCCCTATCCTGC	127bp	
mF9	F: ATGCTGGTGCCAAGTTGGATT<br>R: CTCAGTGCAGGAACAAATTACCT	130bp	387158a1
mApoc3	F: TACAGGGCTACATGGAACAAGC<br>R: CAGGGATCTGAAGTGATTGTCC	116bp	15421856a1
mGBA	F: GTATGGCCTAAGATTCTGGGC<br>R: CTAGGTCACGGGAAATGAAGTC	128bp	118130967c3
mGLA	F: TCTGTGAGCTTGCGCTTTGT<br>R: GCAGTCAAGGTTGCACATGAAA	137bp	7305089a1
mHP	F: GCTATGTGGAGCACTTGGTTC<br>R: CACCCATTGCTTCTCGTCGTT	101bp	8850219a1
mIDS	F: CGGCAGCTTAGCTTCAGCTT<br>R: GAGCATCTGTAGCAGAGTTGC	85bp	23268604a1
mIDUA	F: GCTGACCAGTACGACCTTAGT<br>R: TACGGCACCTATGTAGGCAAG	60bp	6680349a1
